# Clinical Efficacy and Metabolomics Modifications Induced by Polyphenol Compound Supplementation in the Treatment of Residual Dizziness following Semont Maneuver in Benign Paroxysmal Positional Vertigo (BPPV) of the Posterior Semicircular Canal (PSC): Preliminary Results

**DOI:** 10.3390/metabo14020086

**Published:** 2024-01-25

**Authors:** Augusto Pietro Casani, Roberto Albera, Cristina Piras, Andrea Albera, Antonio Noto, Nicola Ducci, Luigi Atzori, Sergio Lucisano, Michele Mussap, Vassilios Fanos

**Affiliations:** 1 Department of Medical and Surgical Pathology, Otorhinolaryngology Section, Pisa University Hospital, 56024 Pisa, Italy; 29550320@studenti.unipi.it; 2 Department of Surgical Sciences, University of Turin, 10024 Turin, Italy; roberto.albera@unito.it (R.A.); a.albera@unito.it (A.A.); slucisano@cittadellasalute.to.it (S.L.); 3 Department of Biomedical Sciences, University of Cagliari, 09121 Cagliari, Italy; cristina.piras@unica.it (C.P.); antonionoto@hotmail.com (A.N.); latzori@unica.it (L.A.); 4 Department of Surgical Sciences, University of Cagliari, 09121 Cagliari, Italy; michele.mussap@unica.it (M.M.); vafanos@tiscali.it (V.F.)

**Keywords:** metabolomics, metabolites, precision medicine, laboratory medicine, polyphenol, residual dizziness, benign paroxysmal positional vertigo, vertigo, Semont maneuver

## Abstract

Benign paroxysmal positional vertigo (BPPV) represents the most frequent cause of peripheral vertigo. In most cases, it is successfully treated using the canalith repositioning procedure, but it is often followed by continuous lightheadedness in the absence of vertigo or nystagmus (residual dizziness, RD). Our aim is to describe the clinical effectiveness and the urine metabolomics profile of treating these patients with polyphenol compound supplementation. We enrolled 30 patients reporting RD after BPPV of the posterior semicircular canal (PSC) successfully treated using the Semont maneuver. Supplementation with a polyphenol compound was administered for 60 days, and patients were evaluated after 30 and 60 days of treatment using self-administered questionnaires (Visual Analog Scales for Dizziness and Nausea, Dizziness Handicap Inventory, DHI) and urine metabolomics analysis performed using 1H-NMR spectroscopy and multivariate followed by univariate analysis. Most patients reported excellent or good efficacy in the treatment of RD with a significant decrease in VAS and DHI values. The metabolomics analysis identified six significant metabolites related to the treatment, namely 1-methylnicotinamide, anserine, hippurate, lysine, methyl succinate and urea, indicating the inflammatory activities and antioxidant properties of the polyphenol compound. These preliminary data suggest that supplementation with a polyphenol compound could induce some metabolic changes that can help in recovery from RD. However, future steps will require confirmation with a more significant cohort of patients and an extension of the metabolomics evaluation to other problems concerning the different clinical aspects of BPPV, such as the high rate of relapse.

## 1. Introduction

Benign paroxysmal positional vertigo (BPPV) is the most common peripheral vestibular disorder [1], with an overall prevalence of 2.4% in the adult general population [2]; BPPV is characterized by paroxysms of vertigo triggered by head position changes in the direction of gravity. BPPV is explained by the migration of degenerated otoconia into the semicircular canals, with the posterior semicircular canal (PSC) being the most commonly involved due to its position in the inner ear [3]. The presence of a critical mass of otoconia moving in the endolymph within the semicircular canals, as the head changes position with respect to gravity, induces a flow that stimulates the cupula, inducing positional nystagmus and vertigo [4]. Most cases of BPPV are idiopathic in origin and probably result from degeneration of the macula. Secondary causes of BPPV refer to identifiable causes of otoconial dislodgement including otologic and non-otologic surgery, head trauma and inner ear disorders which ultimately lead to the degradation of otoconia (vestibular neuritis, Ménière’s disease and sudden sensorineural hearing loss) [5,6]. Although BPPV can usually be resolved without special treatment, the canalith repositioning procedure (CRP) can promote recovery from BPPV [1,2,3,4,7,8]. CRP can help patients gain relief from BPPV by moving the otoconia towards the utriculus, where they are reabsorbed or dissolved. Successful CRP means that nystagmus (negative Dix–Hallpike test results for PSC-BPPV and negative supine roll test for horizontal semicircular canal BPPV) and vertigo symptoms disappear after CRP [1,4,7]. However, after a seemingly successful physical treatment, up to two-thirds of patients are still dizzy and off-balance (residual dizziness, RD) [9,10,11]. RD seems to be a syndrome caused by multiple factors (age, female gender, secondary BPPV, a longer duration of BPPV before treatment, higher Dizziness Handicap Index (DHI) score before treatment, anxiety, osteopenia [11,12]) whose early recognition could contribute to the prevention and treatment of RD. A recent paper indicated that age, course of disease, number of maneuvers, anxiety state, diabetes mellitus, and hypertension were independent risk factors affecting the treatment outcomes of RD after cured BPPV [13]. Among the several tests which can be applied to better understand the pathophysiology of a disease, metabolomics represents a promising possibility in describing metabolic modifications during an illness and after a therapeutic intervention, which can subsequently help to elucidate the mechanisms underlying some biological processes [14]. Metabolomics focuses on measuring low-molecular-weight metabolites, which are either metabolic intermediates or metabolic end-products resulting from cellular metabolism, in biological specimens [15,16]. Most biological specimens studied in metabolomics include blood samples (either serum or plasma), saliva and urine [17]. To date, metabolomics is considered a research tool that can give novel insights in the diagnosis, treatment and prognosis of ENT diseases, such as head and neck cancers, allergic rhinitis, sudden sensorineural hearing loss, noise-induced hearing loss and Menière’s disease [18,19,20]. Despite its high clinical incidence, to date, no metabolomics studies have been conducted on BPPV and on residual dizziness after successful treatment with CRM. Several medications have been proposed as approaches for RD, but the results reported are conflicting. Recently, good therapeutic results were obtained using supplementation with a nutraceutical (Vertigoval^®^, Valeas, Italy), which was proven to be able to reduce subjective symptoms and improve instability earlier [21], decreasing the risk of potential complications such as an increased risk of falls, activity restrictions and social and economic burdens, especially in the elderly, and in patients with psychiatric comorbidities [22,23]. The components of Vertigoval^®^ are citicoline and vitamin B6 (active in facilitation of the function of the central vestibular system, having a protective effect on microvascular circulation [24]); Melissa (able to potentiate gamma-aminobutyric acid (GABA) receptors, alleviating stress, which has been counted among the conditions underlying RD [25]); ginger (having an antiemetic power, known since antiquity) [26]; and ViNitrox, a synergistic combination of apple and grape polyphenols that has been demonstrated to have a remarkable vasodilator effect through endothelial nitric oxide synthase (NOS) and antioxidant activity [27,28].

The present study aims to correlate the metabolic profiles of patients suffering from RD treated for 60 days with supplementation with a nutraceutical (Vertigoval^®^) containing ViNitrox, vitamin B6, citicoline, Melissa officinalis and ginger, and its main objectives are to investigate a possible urine metabolic profile in patients suffering from RD after successful treatment of PSC-BPPV and to improve medical knowledge on pathophysiology of residual dizziness.

## 2. Materials and Methods

### 2.1. Participants

The enrolled population included 30 participants (18 females and 12 males), aged (mean ± SD years) 56.1 ± 15.6, consecutively admitted to the two tertiary referral centers involved in the study (17 patients from the Department of Otolaryngology of the University Hospital of Torino and 13 patients from the Department of Otolaryngology of the University Hospital of Pisa). Each patient suffered from BPPV due to the involvement of the PSC and was successfully treated with the Semont maneuver [7]. PSC-BPPV was diagnosed according to the Bàràny Society criteria [29]. The affected side was the right in 17 cases (57%) and the left in 13 cases (43%). The positive result of the Semont maneuver was evaluated 2 or 3 days after the treatment (at the next follow-up in the outpatient department) based on the absence of vertigo and nystagmus while performing the Dix–Hallpike test. Inclusion criteria were the presence of dizziness (RD) eventually associated with neurovegetative symptoms (mainly nausea) after canalolithiasis of PSC successfully treated with CRP. We excluded patients with secondary BPPV, central nervous system diseases, alcohol or psychoactive substance abuse, uncontrolled hypertension, hypotension, thyroid diseases, severe coronary artery disease, severe renal or hepatic failure, pregnancy or breast-feeding, chronic instability pre-existing at the onset of BPPV, known intolerance to polyphenol compound supplementation or poor compliance.

### 2.2. Subjective Symptom Assessement

The subjective symptomatology at diagnosis (T0) and its modifications after polyphenol compound therapy (1 tablet 2 times a day for 60 days) were studied by means of the Visuo-Analog Scale (VAS) [30] and of the Italian version of the Dizziness Handicap Inventory (DHI) [31]. The Visuo-Analog Scale (VAS) is aimed at quantifying the intensity of dizziness. Patients were asked to indicate on a 100 mm long line their subjective “level” of intensity of symptoms at T0 (basal), T30 (after 30 days of treatment) and T60 (after 60 days of treatment). The Dizziness Handicap Inventory (DHI) is a self-administered questionnaire that estimates the impact of balance disorders on the quality of life (QoL) [32]. It consists of 25 questions regarding the emotional (9 questions), functional (9 questions) and physical (7 questions) domains of daily life. To each question, the patient could answer with a number ranging from 0 to 4, with 0 meaning “No”, 2 meaning “Sometimes” and 4 meaning “Yes”. The scoring of the results is performed in the following way: 0–14 points indicate a normal handicap, 16–34 points indicate a mild handicap, 36–52 points indicate a moderate handicap and a score over 54 points severe QoL worsening. [33,34]. Like the VAS Dizziness scale, the DHI was also administered during the initial patient evaluation (T0—basal) and then again after 30 (T30) and 60 (T60) days of treatment with polyphenol compound supplementation (Vertigoval^®^). In addition to objective outcome measures, in order to claim the status of the main outcome measure, we asked patients about the efficacy (relief of symptoms) and tolerability (patient perspective of adverse drug reactions) of the treatment, reported as excellent, good, sufficient or poor (patient-oriented outcome measures) [35,36].

### 2.3. Urine Sample Preparation and ^1^H-NMR Analysis

Urine collection was performed at T0, T30 and T60 with clinical evaluation. An aliquot of 800 µL of urine was transferred into an Eppendorf tube with 8 µL of a 1% aqueous solution of NaN_3_ to inhibit bacteria growth and stored at −80 °C. Before the analysis, the sample was centrifuged at 12,000× *g* for 10 min at 4 °C to remove solid particles. Then, 630 µL of the supernatant was mixed with 70 µL of potassium phosphate buffer in D_2_O (1.5 M, pH 7.4) containing sodium 3-trimethylsilyl-propionate-2,2,3,3,-d4 (TSP) as an internal standard (98 atom% D, Sigma-Aldrich, Milan, Italy). Finally, an aliquot of 650 µL was transferred to 5 mm NMR glass tubes for 1H-NMR analysis. NMR analysis was carried out using a Varian UNITY INOVA 500 spectrometer operating at 499.839 MHz for proton NMR and a 5 mm double resonance probe (Agilent Technologies, Santa Clara, CA, USA). One-dimensional proton NMR spectra were obtained using a 1D nuclear Overhauser enhancement spectroscopy (NOESY) standard pulse sequence to suppress the signal of water with a relaxation delay of 3 s. For each sample, 256 free induction decays (FIDs) were collected into 64 K data points with a spectral width of 6000 Hz with a 90° pulse, an acquisition time of 2 s and a mixing time of 100 ms. The FIDs were weighted using an exponential function with a 0.5 Hz line-broadening factor prior to Fourier transformation.

#### H-NMR Data Preprocessing

NMR spectra were phased and baseline-corrected using ACD/Lab Processor Academic Edition (Advanced Chemistry Development, 12.01, 2010) and chemical shifts referenced internally to TSP at d = 0.0 ppm. Next, the spectral region comprising the signal of residual water (4.7–4.9 ppm) was removed. The final spectral regions considered were in the ranges of 0.7–4.7 ppm and 4.9–9.5 ppm. The ACD Labs intelligent bucketing method was used for spectral integration (20). A 0.04 ppm bucket width was defined with an allowed 50% looseness. The degree of looseness allows the bucket width to vary over a particular value from the set bucket value. The intelligent bucket method identifies local minima in the spectra and sets the buckets accordingly. In this manner, a peak is integrated into one bucket, although there may be minor chemical shift differences due to pH, for instance.

The area of bucketed regions was normalized using median fold change normalization (MFC) [37], primarily preferred to total sum normalization when studying urine samples. Finally, the spectral data were imported into the SIMCA software (Version 16.0, Sartorius Stedim Biotech, Umea, Sweden) for multivariate statistical analysis. All imported data were then preprocessed using Pareto scaling [38].

### 2.4. Statistical Analysis

For each medical examination, the results of DHI and VAS were collected, and the mean and SD were calculated (mean ± SD). For T30 and T60, mean values were correlated with basal (T0) mean values by calculating their percentual variation (Δ%). A comparison of T30 and T60 with respect to their basal values was performed by means of the Student’s *t*-test. For each medical examination, the numerical answers to all the 25 questions of DHI were added, and the mean and SD were calculated. Moreover, the mean and SD for the questions related to each of the three domains (emotional, functional and physical) were calculated. The mean values recorded at T30 and T60 were also compared to the basal (T0) values by calculating their percentual variation (Δ%). A comparison of T30 and T60 values with respect to their basal values was performed by means of the Student’s *t*-test.

The multivariate statistical analysis was based on principal component analysis (PCA, Addinsoft by Lumivero, Denver, CO, USA) and orthogonal partial least square discriminant analysis (OPLS-DA). PCA, an unsupervised pattern recognition method, was performed to evaluate the samples’ homogeneity at T0, T30 and T60 and identify any possible trends or outliers (defined as observations located outside the 95% confidence region of the model) [39]. PCA is an unsupervised pattern recognition method, and its purpose is to express the main information contained in the initial variables in a lower number of uncorrelated variables, the so-called principal components (PCs), which are combinations of the initial measurements but highlight the variance within the dataset and remove redundancies. Successive PCs account for decreasing amounts of variance, and most of the information is contained in the first few PCs. Graphically, the output from PCA consists of a score plot, giving an indication of any grouping in the datasets in terms of metabolomics similarity, and a loading plot, providing an indication as to which variables are important with respect to the patterns obtained in the score plot.

OPLS-DA, a supervised method, was used to reduce model complexity and highlight sample discrimination [14]. The quality of the OPLS-DA model was evaluated using a 7-fold cross-validation and permutation test (500 times). OPLS-DA is a supervised classification technique and maximizes the covariance between the measured data of the X-variable (peak intensities in NMR spectra) and the response of the Y-variable (class assignment) within the groups. The permutation test was calculated by randomizing the Y-matrix (class assignment or continuous variables) while the X-matrix (peak intensity in NMR spectra) was kept constant. The permutation plot then displayed the correlation coefficient between the original y-variable and the permuted y-variable on the x-axis versus the cumulative R2 and Q2 on the y-axis and included a regression line. The intercept is a measure of the overfit; a Q2Y intercept value less than 0.05 indicates a valid model. The estimated predictive power of the models was expressed by R2Y and Q2Y, which represent the fraction of the variation in the Y-variable and the predicted fraction of the variation in the Y-variable, respectively. A good prediction model is achieved when Q2 ≥ 0.5. One-way analysis of variance (ANOVA) and Kruskal–Wallis tests were performed to reveal the significance of differences between groups. To control the false-discovery rate, Benjamini and Hochberg’s procedure was applied (with the Benjamini–Hochberg FDR of 0.05).

## 3. Results

### 3.1. Visuo-Analog Scale (VAS) of Dizziness

Mean VAS Dizziness values are reported in Table 1. Table 1 depicts mean value changes in the VAS Dizziness scale at T30 and T60 (at 30 and 60 days of treatment with Vertigoval^®^, respectively) in comparison with the basal level (T0). According to the VAS values, a statistically significant reduction in dizziness was detected in all the patients treated with the polyphenol compound. The reduction in the VAS Dizziness value was 48.8% and 82.5% at T30 and T60, respectively, compared to T0.

### 3.2. Dizziness Handicap Inventory (DHI)

**Figure 1:**
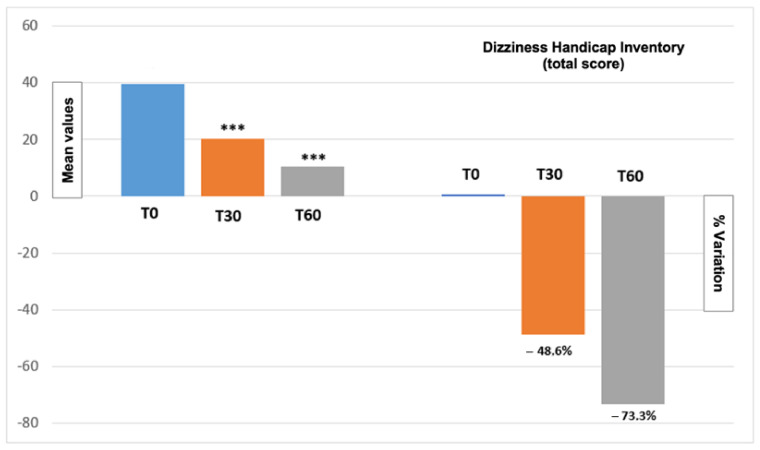
Mean values and percentual variation of total DHI score at T0 (basal), T30 (after 30 days of treatment) and T60 (after 60 days of treatment) in the 30 treated patients. After 30 (T30) and 60 (T60) days of treatment, a markedly lower scoring was obtained (−48.6% and −73.3%, respectively) with a statistically significant variation (*** *p*-value < 0.0001).

**Figure 2:**
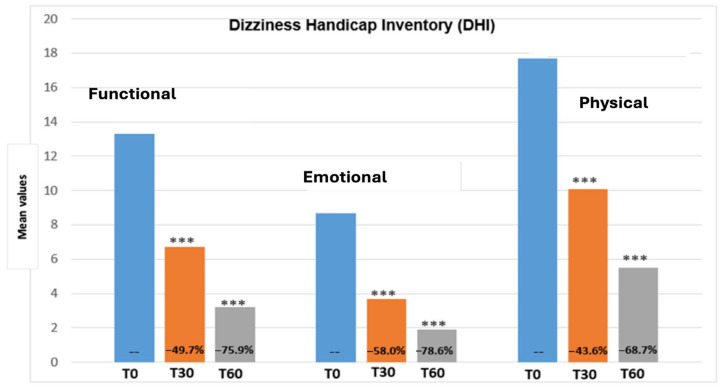
Scores obtained on the three DHI subscales (functional, emotional and physical) at T0 (basal), T30 (after 30 days of treatment) and T60 (after 60 days of treatment) in the 30 treated subjects. Results of statistical variation comparison using Student’s *t*-test are indicated (*** *p*-value < 0.0001).

Figure 1 reports the total DHI score variation from the beginning until the end of the treatment period. The total DHI score showed a significant decrease at 30 and 60 days post-treatment (*p* < 0.0001) with a T30 and T60 reduction of −48.6% and −73%, respectively. Figure 2 shows values regarding the three DHI subscales (emotional, functional and physical). All the DHI subscales values showed a marked reduction with no clear differences between the functional (T30 −49.7%, T60 −75.9%), emotional (T30 −58%, T60 −78.6%) and physical (T30 −43.6%, T60 −68.7%) subscales. The progressive reduction in the DHI value demonstrated a significant impact on health-related quality of life (QoL).

### 3.3. Efficacy and Tolerability Evaluation

After 30 and 60 days of treatment, both the clinician and the patient were asked to express their opinion regarding the overall efficacy and tolerability of the medication. The efficacy and tolerability of the polyphenol compound were reported as excellent or good in 100% of the patients at T30 and T60.

### 3.4. Urine Metabolomics

The urine metabolomics profile of samples at T0, T30 and T60 was investigated using ^1^H-NMR spectroscopy coupled with multivariate statistical analysis. Metabolites were identified based on the literature [39] and using dedicated libraries, such as the Human Metabolome Database (HMDB, http://www.hmdb.ca accessed on 11 January 2022) and the 500 MHz libraries from Chenomx NMR suite 7.1. A first PCA as the samples’ homogeneity. Only one sample at a time, T60, was excluded from subsequent analyses.

**Figure 3:**
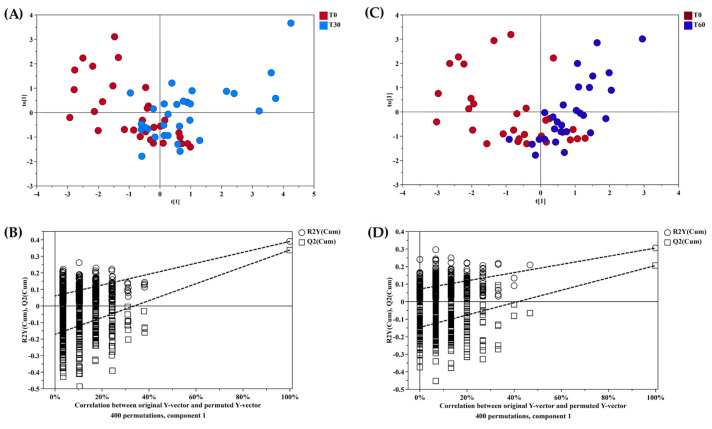
(**A**,**C**) OPLS-DA score plots of ^1^H-NMR spectra of urine samples. Red dots: T0; light blue dots: T30; blue dots: T60. (**B**,**D**) Permutation test. The horizontal axis shows the correlation between the permuted and actual data, while the vertical axis displays the cumulative values of R2 and Q2. The intercept gives an estimate of the overfitting phenomenon.

**Figure 4:**
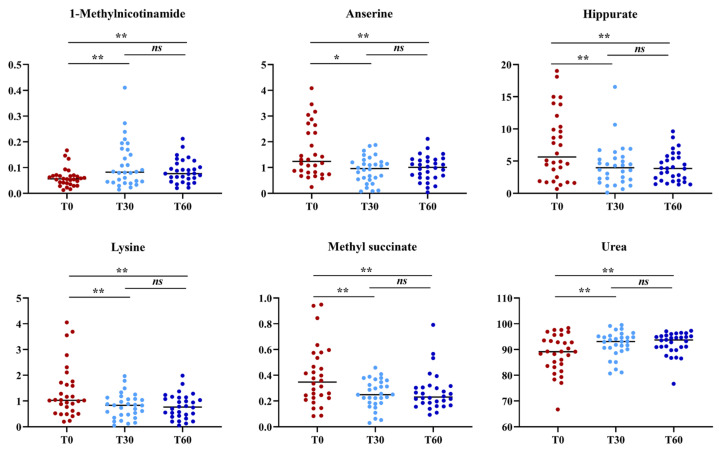
Scatter plots showing the progressive change in the urine metabolite levels in the samples at different times (T0, T30 and T60). Statistical significance was determined using one-way analysis of variance (ANOVA) and Kruskal–Wallis tests. * *p*-value ≤ 0.01 and ** *p*-value < 0.05. *ns*: not significant.

Then, a supervised statistical analysis was performed on the same dataset. The statistical analysis on the whole urinary profile was not able to discriminate the samples analyzed at T0 with respect to the subsequent analysis times. This may be due to an unidentified number of disturbing variables and/or noise variables that characterize the NMR spectra. Thus, a univariate statistical analysis was performed on the different spectral regions identified with the intelligent bucket method to remove potential information unrelated to the pathology. Only the spectral regions with a *p*-value < 0.05 were quantified with Chenomx NMR suite 7.1. The spectral regions with a *p*-value < 0.05 were characterized by metabolites such as 1-methylnicotinamide, anserine, hippurate, lysine, methyl succinate and urea. Then, a new statistical supervised model was created using only the six identified metabolites. In Figure 3A, the OPLS-DA model shows how the samples at time T0 are partly separated along t(1) with respect to T30, but a large number of samples overlap in the intersection of t(1) with t(2). The OPLS-DA model was established with one predictive and one orthogonal component and showed R2X, R2Y and Q2 values of 0.613, 0.306 and 0.206, respectively. The validity of the OPLS-DA model was tested through a permutation test (Figure 3B) using 400 times. R2 and Q2 intercept values of 0.07 and −0.14, respectively, indicate a valid model. The OPLS-DA model in Figure 3C shows how the samples at time T60 are more separated along t(1) than at T0, indicating how the polyphenol compound supplement after two months of treatment has a more significant impact on the metabolomics profile. The OPLS-DA model was established with one predictive and one orthogonal component and showed R2X, R2Y and Q2 values of 0.598, 0.390 and 0.339, respectively. The validity of the OPLS-DA model was tested through a permutation test (Figure 3D) using 400 times. R2 and Q2 intercept values of 0.06 and −0.17, respectively, indicate a valid model. The concentration variation of the six discriminant metabolites in the samples at different times was evaluated with one-way analysis of variance (ANOVA) and Kruskal–Wallis tests. The univariate statistical analysis results are shown using scatter plots (Figure 4). The subjects at T30 and T60 were characterized by a higher level of 1-methylnicotinamide and urea and lower levels of anserine, hippurate, lysine and methyl succinate compared to T0 (Table 2).

## 4. Discussion

RD is a common experience that manifests as persistent imbalance after successful CRP for BPPV. These symptoms may affect the quality of life and prevent carrying out daily activities. The decreasing postural control can contribute to falling and psychological problems [40]. The prevalence of these residual symptoms varies and depends on the patient’s description of their symptoms and the physical handicap perceived by the patient. Previous studies reported an association with a condition of stress of the subject that may be correlated with the duration of the BPPV and the number of recurrences [41,42]. Some authors reported the duration of the residual symptoms was about 6–20 days [10,12,41,42,43]. However, some patients recover later than others, and some of these still present symptoms after 1 and 2 months [12,40,43]. The decreased postural control can contribute to falling and psychological problems [44]. Although many hypotheses have been postulated, the origin of this disturbance is not yet clear [10,11,12,45]. Several medications have been proposed to approach these symptoms [46,47,48], but nothing has yet proved to provide relief for residual dizziness compared to placebo. The most used molecule to approach residual dizziness after BPPV is betahistine dihydrochloride, but its results are conflicting [49,50]. Recently, a multicentric non-double-blinded designed study reported a statistically significant decrease in DHI and VAS values after 60-day supplementation with a polyphenol compound that proved to be able to reduce subjective symptoms and improve instability earlier, decreasing the risk of potential complications without any significant side effects [21].

In order to confirm the effective validity of the treatment of RD we propose, we studied the metabolomics aspects in a group of patients treated with polyphenol supplementation in an effort to correlate the positive clinical results with those of a metabolomics study. Using a metabolomics approach, it is possible to obtain detailed information to understand the molecular phenotype of a particular disease. This approach generates a plethora of data, which can help to understand the mechanisms underlying biological processes and molecular functions [14,15]. Phenomena in vestibular and auditory neuroscience, as in other areas of neuroscience, involve the complex interaction of multiple variables. MVAs and data mining methods have been successfully used in metabolomics studies to predict the progression of diseases [14,16,39,51,52]. Thus, applying a 1H-NMR analysis coupled with an MVA approach to BPPV patients treated for 60 days with a nutraceutical (Vertigoval^®^), we aimed to identify biomarkers related to the treatment. The metabolomics analysis identified six significant metabolites: 1-methylnicotinamide, anserine, hippurate, lysine, methyl-succinate and urea. Urea and 1-methyl-nicotinamide showed an increasing trend from T0 to T60, while anserine, hippurate, lysine, and methyl-succinate resulted in a reduction. Urea is synthesized in the urea cycle from ammonia or by the oxidation of amino acids; thus, it is the main product of protein catabolism. Urea production occurs mainly in the liver and, to a lesser extent, in the kidneys, and an increased concentration may reflect protein catabolism which may increase due to inflammatory processes [53]. In contrast, the increased concentration of 1-methyl-nicotinamide at 30 and 60 days appears to be related to the anti-inflammatory property of this molecule. Lakshmi and Bamji [54] have suggested evaluating urinary 1-methylnicotinamide to indirectly determine niacin (Vitamin B3) status, which is of great importance for monitoring the health of energy metabolism. Normal adults excrete more than 17 μmol 1-methyl-nicotinamide per day, whereas during deficiency, its excretion is less than 5.8 μmol per day. Patients treated for two months with the nutraceutical (Vertigoval^®^) had a statistically increased level of 1-methyl-nicotinamide, which could be interpreted as a restoration of the level of niacin and nicotinamide adenine dinucleotide (NAD+) levels. [55]. The level of niacin is also related to lysine metabolism. Lysine is an alpha-amino acid, and its catabolism occurs through several biochemical pathways. A recent phase 4 clinical study analyzed the correlation between vertigo and lysine, underlying a solid connection (https://www.ehealthme.com/ds/l-lysine/vertigo/ accessed on 12 January 2022). In particular, a high lysine concentration is related to the presence of vertigo, especially in females older than 60 years. Therefore, a significant reduction in lysine concentration after 60 days of nutraceutical (Vertigoval^®^) supplementation could be interpreted as an ameliorant indicator. Anserine is a dipeptide containing beta-alanine and 3-methylhistidine; it is the most common variant of methylated carnosine analogs. Carnosine has several antioxidant properties and has been proven to scavenge reactive oxygen species (ROS) and alpha–beta-unsaturated aldehydes formed from the peroxidation of cell membrane fatty acids during oxidative stress [56]. Patients treated for 60 days with the nutraceutical (Vertigoval^®^) showed a reduction in anserine, which may be interpreted as a reduction in ROS presence. In agreement with this, the level of glutamine has been shown to be related to a reduction in neuronal swelling caused by acoustic trauma [57] via 5-aminovalerate transaminase. A reduction in hippurate, the glycine conjugate of benzoic acid, is interpreted as a plausible hallmark of changes in gut microflora [58]. These results are absolutely in agreement with the favorable clinical outcome. VAS and DHI values showed a progressive and significant reduction in RD at T30 and T60. The congruence between the metabolomics approach and the clinical results can be easily supported by analyzing the characteristics of the components of the nutraceutical we used. Citicoline might facilitate the function of the central vestibular system, activating the biosynthesis of phospholipids in neuronal membranes and consequently stimulating brain metabolism and the neurotransmitter system [59]. Furthermore, citicoline, apart from its well-known neuroprotective effects in acute ischemic stroke [24], potentiates angiogenesis [60] with a consequent protective effect on microvascular circulation. The dose-dependent efficacy of citicoline in the management of central and ischemic vertigo was documented. However, enrolled patients were treated with betahistine too, so it is not easy to evaluate if the clinical efficacy was related to citicoline, betahistine or both [61]. Melissa seems to be able to potentiate gamma-aminobutyric acid (GABA) receptors, alleviating stress, which has been counted among the conditions underlying the residual instability after BPPV, as previously reported [25]. The antiemetic power of ginger, on the other hand, has been known since antiquity. The last component is ViNitrox, a synergistic combination of apple and grape polyphenols that has been demonstrated to have a remarkable vasodilator effect through endothelial nitric oxide synthase (NOS) and antioxidant activity [62]. The combination seems effective in at least partially restoring the microvascular impairment in these patients and also improving cognitive function. Usefully, a recent article published by Ulivi et al. [63] reports that supplementation for 60 days with the nutraceutical we studied reduces oxidative stress load in patients with a pre-existing imbalance, improving dizziness symptoms. Even though the study was carried out on patients suffering from chronic dizziness because of underlying small vessel disease, the mechanisms underlying the symptom’s improvement in this category of patients can be the same as those underlying the ability to grant relief to the patients recruited in our study.

## 5. Conclusions

Our results confirm the positive activity of supplementation with a polyphenol compound in improving the symptomatology of residual dizziness after successful treatment of BPPV, and the metabolomics analysis provides excellent scientific support for this evidence. This is the first preliminary approach to BPPV and RD with metabolomics. The preliminary data from the present study suggest that supplementation with a polyphenol compound could induce some metabolic changes that can help in recovery from RD. However, the future steps will require confirmation with a more significant cohort of patients and an extension of the metabolomics evaluation to other problems concerning the different clinical aspects of BPPV, such as the high relapse rate.

## Figures and Tables

**Table 1:** Mean values, SD (standard deviation) and Δ% (difference in percentage terms) of recorded data for VAS Dizziness scale at T0 (basal), T30 (after 30 days of treatment) and T60 (after 60 days of treatment) in the 30 treated subjects. Results of statistical variation comparison using Student’s *t*-test are indicated (*** with *p*-values < 0.0001).

VAS Dizziness			
	T0 Mean ± SD	T30 Mean ± SD (Δ%)	T60 Mean ± SD (Δ%)
Mean ± SD (30 subjects)	45.3 ± 4.2	23.2 ± 2.9 (−48.8%)	8.0 ± 1.8 (−82.5%)
Student’s *t*-test	-	*p* < 0.0001 ***	*p* < 0.0001 ***

**Table 2:** Relative concentrations of discriminant metabolites in samples at different times (T0, T30 and T60). Data are expressed as median and 25th–75th percentile IQR. ^a^ For each sample, the relative concentration was obtained by normalizing the molar concentration of each metabolite to the total molar concentration of all six metabolites. * Kruskal–Wallis test corrected by Benjamini–Hochberg multiple comparison. ns: not significant.

	T0	T30	T60	T0 vs. T30	T0 vs. T60	T30 vs. T60
Metabolites (mM) ^a^	Median (Interquartile Range)			*p*-Values *		
1-Methylnicotinamide	0.05 (0.03–0.06)	0.08 (0.04–0.17)	0.07 (0.05–0.11)	0.01	0.02	ns
Anserine	1.24 (0.78–2.42)	0.96 (0.53–1.24)	1.00 (0.60–1.31)	0.01	0.03	ns
Hippurate	5.65 (2.35–10.7)	3.97 (2.05–5.50)	3.85 (1.96–5.61)	0.02	0.03	ns
Lysine	1.02 (0.59–1.72)	0.83 (0.43–1.09)	0.76 (0.41–1.13)	0.02	0.02	ns
Methyl succinate	0.34 (0.22–0.549	0.24 (0.18–0.35)	0.23 (0.18–0.30)	0.02	0.01	ns
Urea	89.1 (83.5–94.0)	93.1 (89.8–95.1)	93.6 (90.6–95.5)	0.04	0.02	ns

## Data Availability

The data presented in this study are available on request from the corresponding author. The data are not publicly available due to ethical restrictions.
